# Diaminopurine in Nonenzymatic RNA Template Copying

**DOI:** 10.1021/jacs.4c02560

**Published:** 2024-05-31

**Authors:** Xiwen Jia, Ziyuan Fang, Seohyun Chris Kim, Dian Ding, Lijun Zhou, Jack W. Szostak

**Affiliations:** † Department of Chemistry and Chemical Biology, Harvard University, 12 Oxford Street, Cambridge, Massachusetts 02138, United States; ‡ Department of Molecular Biology and Center for Computational and Integrative Biology, Massachusetts General Hospital, 185 Cambridge Street, Boston, Massachusetts 02114, United States; § Howard Hughes Medical Institute, Department of Chemistry, The University of Chicago, Chicago, Illinois 60637, United States; ∥ Department of Genetics, Harvard Medical School, 77 Avenue Louis Pasteur, Boston, Massachusetts 02115, United States; ⊥ Department of Biochemistry and Biophysics, Perelman School of Medicine, University of Pennsylvania, Philadelphia, Pennsylvania 19104, United States; # Penn Institute for RNA Innovation, University of Pennsylvania, Philadelphia, Pennsylvania 19104, United States

## Abstract

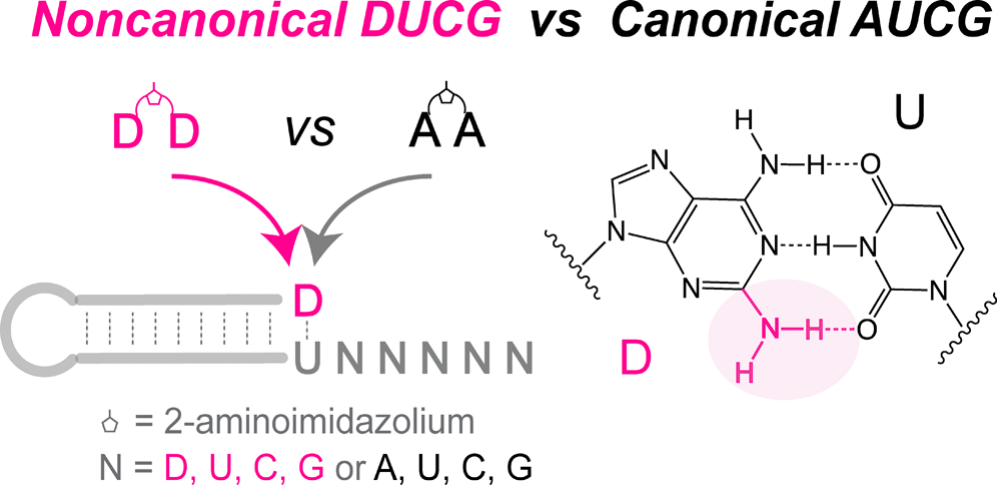

In the RNA World before the emergence of an RNA polymerase, nonenzymatic template copying would have been essential for the transmission of genetic information. However, the products of chemical copying with the canonical nucleotides (A, U, C, and G) are heavily biased toward the incorporation of G and C, which form a more stable base pair than A and U. We therefore asked whether replacing adenine (A) with diaminopurine (D) might lead to more efficient and less biased nonenzymatic template copying by making a stronger version of the A:U pair. As expected, primer extension substrates containing D bound to U in the template more tightly than substrates containing A. However, primer extension with D exhibited elevated reaction rates on a C template, leading to concerns about fidelity. Our crystallographic studies revealed the nature of the D:C mismatch by showing that D can form a wobble-type base pair with C. We then asked whether competition with G would decrease the mismatched primer extension. We performed nonenzymatic primer extension with all four activated nucleotides on randomized RNA templates containing all four letters and used deep sequencing to analyze the products. We found that the DUCG genetic system exhibited a more even product distribution and a lower mismatch frequency than the canonical AUCG system. Furthermore, primer extension is greatly reduced following all mismatches, including the D:C mismatch. Our study suggests that D deserves further attention for its possible role in the RNA World and as a potentially useful component of artificial nonenzymatic RNA replication systems.

## Introduction

The RNA World hypothesis posits RNA as the primordial genetic polymer due to its dual role in encoding information and catalyzing reactions.^1−3^ Prior to the emergence of macromolecular catalysts such as ribozymes, nonenzymatic template copying likely played a critical role in the transmission of hereditary information.^4^ This process, however, has a marked tendency to favor the incorporation of guanosine (G) and cytidine (C) nucleotides over adenosine (A) and uridine (U),^5^ presenting a bias in the copying process. To address this imbalance, we looked beyond the four canonical nucleotides found in RNA, seeking alternatives that could mitigate this issue.

Diaminopurine (D), an adenine analogue characterized by an additional exocyclic amine, is a potentially prebiotic nucleobase. It has been detected in carbonaceous meteorites,^6^ albeit at low parts per billion (ppb) levels. Subsequent studies have demonstrated the synthesis of the D deoxynucleoside (β-2,6-diaminopurine 2′-deoxyriboside) by transglycoslyation of 2-thiouridine with D, although with a low yield of 2%.^7^ A more efficient synthesis of 2,6-diaminopurine ribonucleoside 2′-phosphate from ribose 1′–2′ cyclic phosphate and 2,6-diaminopurine was demonstrated by Kim and Benner,^8^ suggesting that D nucleotides could have formed on the early Earth if a high yielding synthesis of these precursors was possible. Moreover, D and its derivatives, including deoxyribonucleosides and DNA trimers containing D, have a photostability that is only 2-fold less than that of A, implying that D nucleotides could have withstood the UV radiation flux at the surface of the early Earth.^7,9^ Collectively, these findings support the further study of potentially prebiotic pathways for the origin and accumulation of D nucleotides as well as motivating the continued study of potential roles for D in the formation and replication of prebiotic RNA or DNA oligonucleotides.

**Figure 1:**
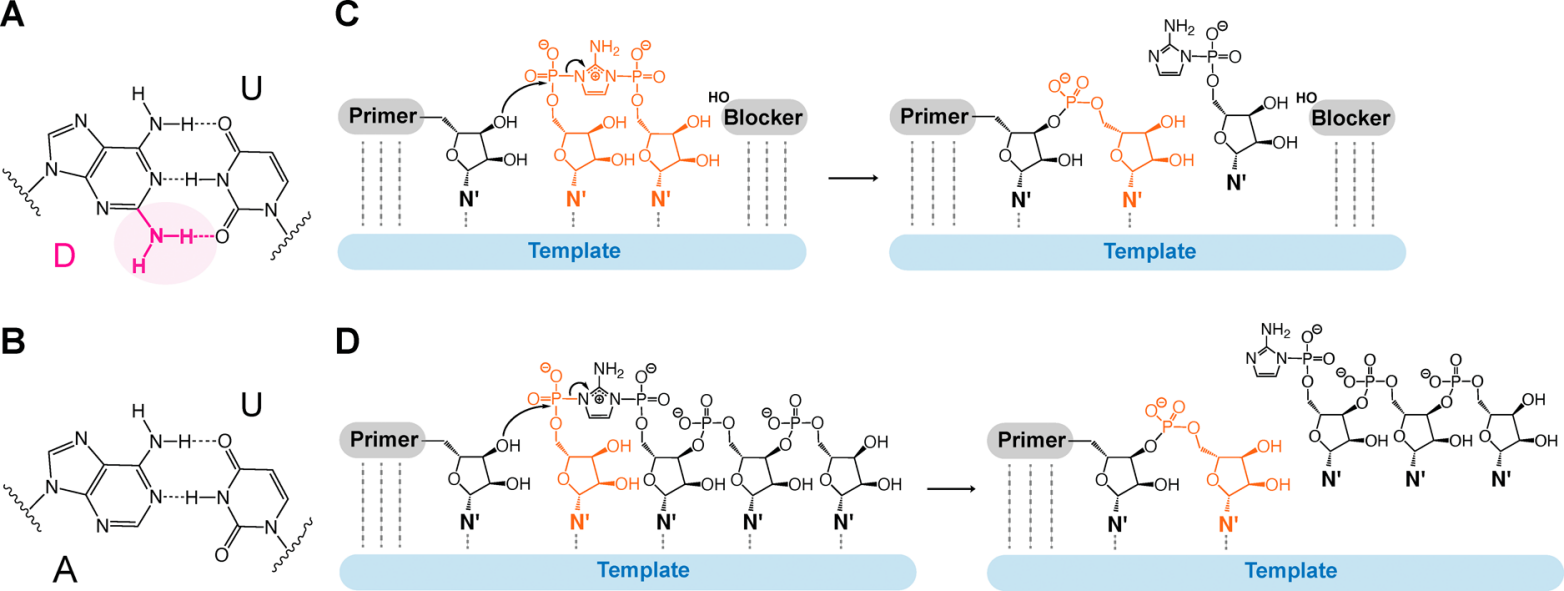
Schematic representation of D:U and A:U base pairs and mechanisms of nonenzymatic primer extension reactions. (A) D:U pair has three hydrogen bonds, whereas the (B) A:U pair has two. Nonenzymatic primer extension can occur through a (C) bridged dinucleotide and a (D) monomer-bridged-trimer intermediate. N′ denotes nucleobase.

In addition to its potential prebiotic relevance, D exhibits molecular properties that may have provided early evolutionary advantages through the stabilization of nucleic acid duplexes. In RNA duplexes, D forms three hydrogen bonds when paired with U, as opposed to two in the A:U pair (Figure 1A,B).^10^ This, together with a stronger stacking interaction, results in a D:U base pair being energetically more favorable than an A:U base pair.^11^ Consequently, there is an increased free energy change upon duplex formation, with a net ΔΔ*G*°_37_ of −0.29 kcal/mol per A:U to D:U base pair substitution, as predicted by a combined molecular dynamics/quantum mechanics (MD/QM) approach.^11^ The larger degree of stabilization is also evidenced by a significant rise in the experimentally determined melting temperature (*T*_m_) of D:U RNA duplexes compared to A:U duplexes.^12^ This enhanced duplex stability extends to other sugar backbones such as DNA^13,14^ and threose nucleic acid.^15^ However, within the context of nonenzymatic RNA replication, the increased duplex stability from the stronger D:U pair may introduce challenges by increasing the difficulty of strand separation.^16^

The potential benefits of D in biological functions have been demonstrated in both RNA and DNA systems, although this substitution has concurrently raised certain concerns. The functionality of RNA with D as a substitution of A is evidenced by the activity of a ribozyme ligase containing only G, D, and U and the in vitro selection of a variant that contains only D and U.^17^ More recently, it has been shown that replacing ATP with DTP enables an RNA polymerase ribozyme to synthesize longer extension products.^18^ Furthermore, D has been used in antisense oligoribonucleotide therapies owing to its duplex stabilizing ability.^19,20^ In DNA, D can fully substitute for A in certain modern biological systems such as cyanophages.^21−23^ This substitution may confer an evolutionary advantage resulting from the ability to evade genome digestion by host restriction enzymes.^22,23^ Additionally, D-substituted DNA probes demonstrate greater selectivity and stronger hybridization to phage or genomic target DNA.^24^ Consequently, D has been harnessed in various biological applications, including antisense DNA technologies^25^ and gene targeting therapies.^26^ However, incorporating D into DNA can undermine the stability of the B-form DNA, sometimes inducing a transition to the Z or A form.^24^ The consequent changes in DNA structure provoke questions regarding the compatibility of D-substituted DNA with the standard DNA-interacting cellular machinery, which might require re-engineering to accommodate D recognition and protein assembly.^27^

Understanding the benefits and challenges of D substitution in RNA and DNA systems motivated us to evaluate the performance of D in nonenzymatic RNA template copying by reviewing prior studies on this topic. The polymerization of imidazole-activated diaminopurine mononucleotides (ImpD) on a polyU template results in longer oligomers compared to ImpA.^28^ Moreover, nonenzymatic copying on an RNA hairpin containing two templating D residues using activated uridine monomers (ImpU) enhances the elongation rates at lower initial concentrations in the eutectic (water-ice) phase.^29^ Other studies using activated mononucleotides with a different activation group, 2-methyl imidazole (2-MeImp), led to similar findings. Replacing an A with a D residue in the middle of a 9-mer DNA template increases the efficiency of U incorporation by 3-fold.^10^ Substituting 2-MeImpA with 2-MeImpD as the substrate allows primer extension to proceed at a much lower concentration, particularly in RNA templates with high U content.^30^ This substitution also yields longer primer extension products on DNA templates containing consecutive thymidine residues.^31^ Taken together, these studies show that D substitution in the template or substrate improves the yield and rate of the nonenzymatic RNA template copying while leaving the fidelity problem unexplored.

Recent advances in nonenzymatic template copying, particularly the discovery of the prebiotically plausible 2-amino imidazole (2AI) activation group^32^ and the highly reactive 5′–5′ imidazolium-bridged intermediates,^33,34^ warrant a re-evaluation of its efficacy. 2AI-activated mononucleotides readily form bridged dinucleotides^35^ that bind to the template through two Watson–Crick base pairs (Figure 1C) and are the predominant contributors to template-directed primer extension.^33^ Mononucleotides can also react with activated trimers to form monomer-bridged-trimer intermediates with enhanced template affinity and faster rates of primer extension (Figure 1D).^34^ In both scenarios, the 3′-hydroxyl group of the terminal nucleotide of the primer attacks the activated substrate, resulting in the primer being extended by one nucleotide (+1 primer extension) with an activated mononucleotide or an activated trimer as the leaving group (Figure 1C,D). Moreover, the recent development of a deep sequencing protocol, Nonenzymatic RNA Primer Extension Sequencing (NERPE-Seq),^36^ enabled us to not only examine the yield but also the fidelity of the DUCG system in the context of nonenzymatic copying. We therefore reassessed the effect of D in these improved nonenzymatic copying systems.

In this study, we report the enhanced affinity of D-bridged dinucleotides for a −UU– template. We then compare the rates of nonenzymatic primer extension in the noncanonical DUCG system to those in the canonical AUCG system. While the D:C mismatch has an unexpectedly elevated reaction rate, the misincorporation of D opposite C hinders the addition of the next nucleotide. Moreover, crystallographic studies show that the D:C pair forms only two hydrogen bonds compared to the three found in the D:U pair. Furthermore, through competition experiments analyzed by next-generation sequencing, we gauge the performance of both the canonical and noncanonical systems under more realistic prebiotic conditions, where four nucleobases coexist in the nonenzymatic primer extension experiments. Intriguingly, the DUCG system yields a more balanced product base distribution and lowers the mismatch frequency. Moreover, primer extension following mismatches is greatly reduced. Collectively, our findings suggest that D improves nonenzymatic template copying, underscoring its potential as a primordial nucleobase.

## Results

### Enhanced Binding Affinity of D:U Pairs

**Figure 2:**
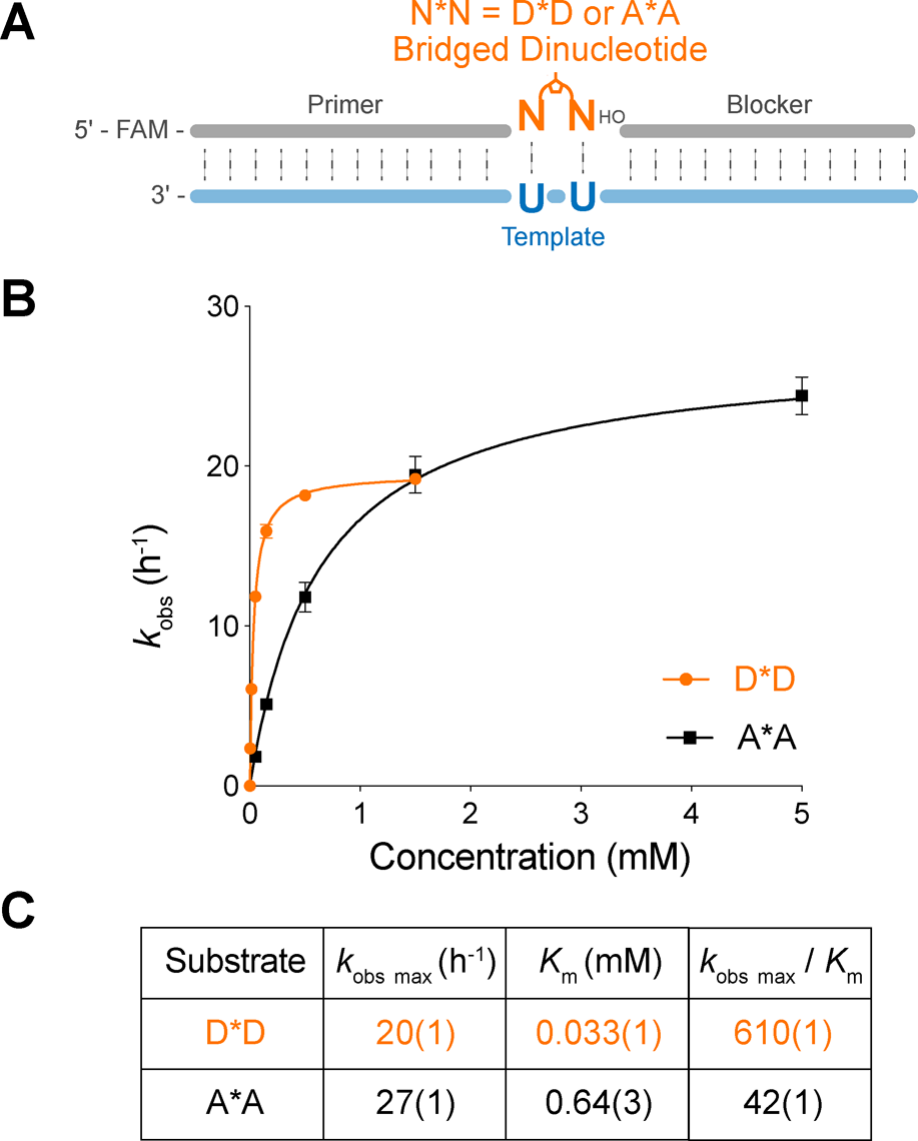
Kinetic study of bridged diaminopurine and adenine dinucleotides D*D and A*A. (A) Schematic representation of the nonenzymatic primer extension reactions. (B) Reaction rate vs concentration curves for D*D and A*A. (C) Observed maximal rates (*k*_obs max_), Michaelis constants (*K*_m_), and their ratios. Primer extension reactions were performed at the indicated concentrations of bridged dinucleotides (D*D or A*A), 1.5 μM primer, 2.5 μM template, 3.5 μM blocker, 100 mM MgCl_2_, and 200 mM Tris at pH 8.0. Error bars and numbers in the parentheses indicate standard deviations of the mean, *n* = 3 replicates. The values of A*A are reproduced from Figure S2 in ref (34) with permission under a Creative Commons Attribution 4.0 International License. Copyright 2022 Oxford University Press.

D forms a more stable pair with uracil compared to adenine, presumably due to the additional hydrogen bond and enhanced stacking interactions.^11^ We sought to investigate the effects of this enhanced affinity on nonenzymatic template copying. To study this, we employed a primer-template-blocker system^34^ with a 2-nt open template region for the bridged-dinucleotide substrate to bind and react (Figure 2A and Table S1). Compared to previous studies of D on nonenzymatic template copying,^10,31^ we employed 5′–5′ imidazolium-bridged intermediates and an RNA template. We measured the pseudo-first-order reaction rate constants (*k*_obs_) for the bridged dinucleotide substrates D*D and A*A as a function of concentration and fitted the data using the Michaelis–Menten equation (Figure 2B). The results indicate that D*D and A*A have similar maximum rates of reaction (*k*_obs max_), which are 20 and 27 h^–1^, respectively. However, the Michaelis–Menten constant (*K*_m_) of D*D is 20-fold lower than that of A*A, 0.033 mM vs 0.64 mM. Taken together, the *k*_obs max_/*K*_m_ of D*D is 15-fold larger than that of A*A, 610 vs 42 (Figure 2C). This finding indicates that the effect of D is greater at lower concentrations.

The pronounced enhancement of the affinity for D*D is in alignment with that predicted by the nearest-neighbor (NN) model. This model predicts the free energy change upon the formation of an RNA double helix, utilizing NN parameters for each stacked pair, a term for the entropy cost of the initial base pairing, and corrections for varied terminal base pairs.^37^ Specifically, the energies associated with D:U stacked pairs and the penalties for the terminal D:U pairs are lower than those of the canonical pairs, resulting in a more negative change in Gibbs free energy value (Table S2).^11,37^ The value of ΔΔ*G*°_37_ predicted by MD/QM modeling, −4.08 ± 0.40 kcal/mol, is in qualitative agreement with our experimental observation, where the *K*_m_ reduction for D*D corresponds to a ΔΔ*G*°_25_ of −1.76 kcal/mol (Supporting Information Discussion section). The discrepancy of these values may be attributed to the temperature variations, approximated coaxial stacking, and the nontrivial effect of 2-aminoimidazole moieties in bridged dinucleotides, which are modeled as dimers. Despite these differences, both our experimental values and the MD/QM predictions indicate a stronger binding affinity of D:U pairs, likely due to the additional hydrogen bond and enhanced stacking interactions (Table S3).^11^

### Kinetic Study of the DUCG System

**Figure 3:**
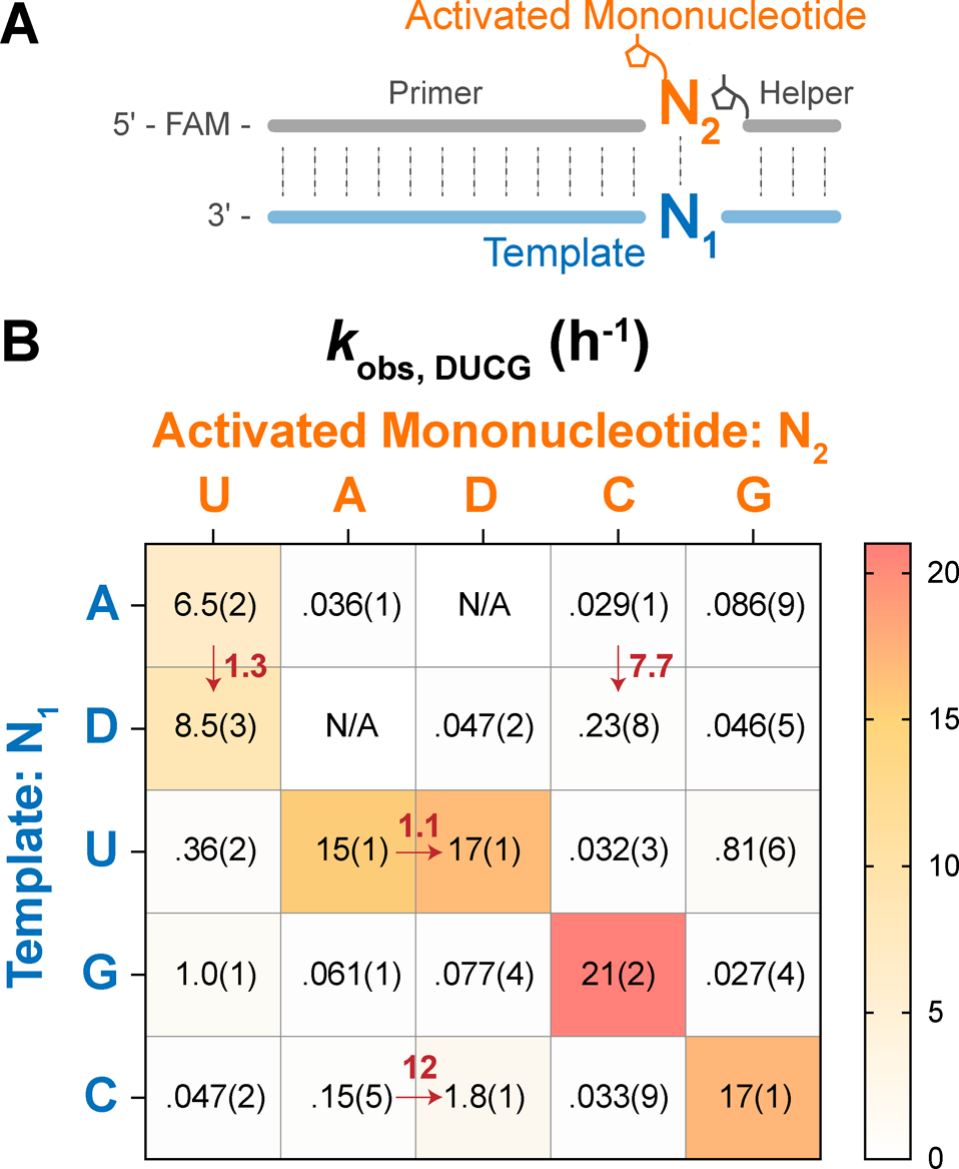
Kinetic study of the DUCG system. (A) Schematic representation of the nonenzymatic primer extension reactions. (B) *k*_obs_ of the primer extension reactions in different combinations of template: N_1_ and the activated mononucleotide: N_2_. Notable rate changes are denoted with arrows pointing to the direction of these changes. Numbers in parentheses indicate standard deviations of the mean, *n* = 3 replicates. Primer extension reactions were performed at 20 mM activated mononucleotides, 0.5 mM activated trinucleotide helper, 1.5 μM primer, 2.5 μM template, 100 mM MgCl_2_, and 200 mM Tris at pH 8.0. Data of the AUCG systems are adapted from Figure 3 in ref (38) with permission under a Creative Commons Attribution 4.0 International License. Copyright 2024 American Chemical Society.

We examined the impact of substituting D for A on nonenzymatic template-directed primer extension by measuring the rates of primer extension for a complete matrix of 2AI-activated mononucleotides (*A, *D, *U, *C, and *G) and template nucleotides (A, D, U, C, and G) (Figure 3A and Table S4). Because primer extension occurs primarily through an imidazolium-bridged intermediate, we used an activated trinucleotide downstream helper (*GAC), which reacts with an activated mononucleotide to form a highly reactive monomer-bridged-trimer intermediate (N*GAC) (Figure 1D).^34^ With 20 mM activated monomer and 0.5 mM activated trimer, the template is essentially saturated with N*GAC at all times. Although the formation of the bridged intermediate and subsequent primer extension reactions are two distinct stages within this model system, the first step is relatively fast. Therefore, we could measure pseudo-first-order reaction rate constants to estimate the efficiency of nonenzymatic primer extension. Under these conditions, primer extension with U is slightly faster on a D template than on an A template. Conversely, the incorporation of a D monomer is very slightly faster than that of an A monomer on a U template (Figure 3B).

Using the above experimental construct, we were also able to measure all mismatched primer extension rates. However, we observed a significant amount of primer-trimer ligation when the N_1_ nucleotide in the template is C. This issue arises because *GAC competes with *N for substrate binding, which interferes with the accurate measurement of the rate of mismatched primer extension. To address this problem, we used a modified template with a CUCC overhang and a *AGG trinucleotide helper to resolve the competitive binding issue with *N (Table S4). We deliberately chose *AGG with a 5′-purine base for a similar stacking interaction between the activated mononucleotide and trinucleotide. This sequence modification enabled more accurate rate measurements of mismatched primer extension. However, it is important to note that the associated downstream template and helper sequences are different from the standard construct in this case.

When we examined the primer extension rates for mismatched base pairs, we were surprised to see that the reaction rates for the D:C mismatch are markedly higher than those of the A:C mismatch (12-fold for N_1_:N_2_ = C:D and 7.7-fold for N_1_:N_2_ = D:C). This increase in reaction rates for D:C pairs raises concerns regarding the fidelity of the DUCG system. To investigate the consequences of enhanced D:C mismatch formation, we measured its stalling effect. In addition, we solved the crystal structures of RNA duplexes containing D:C pairs to help understand their increased formation rate.

### D:C Mismatches

#### Stalling Effect of D:C Mismatches

**Figure 4:**
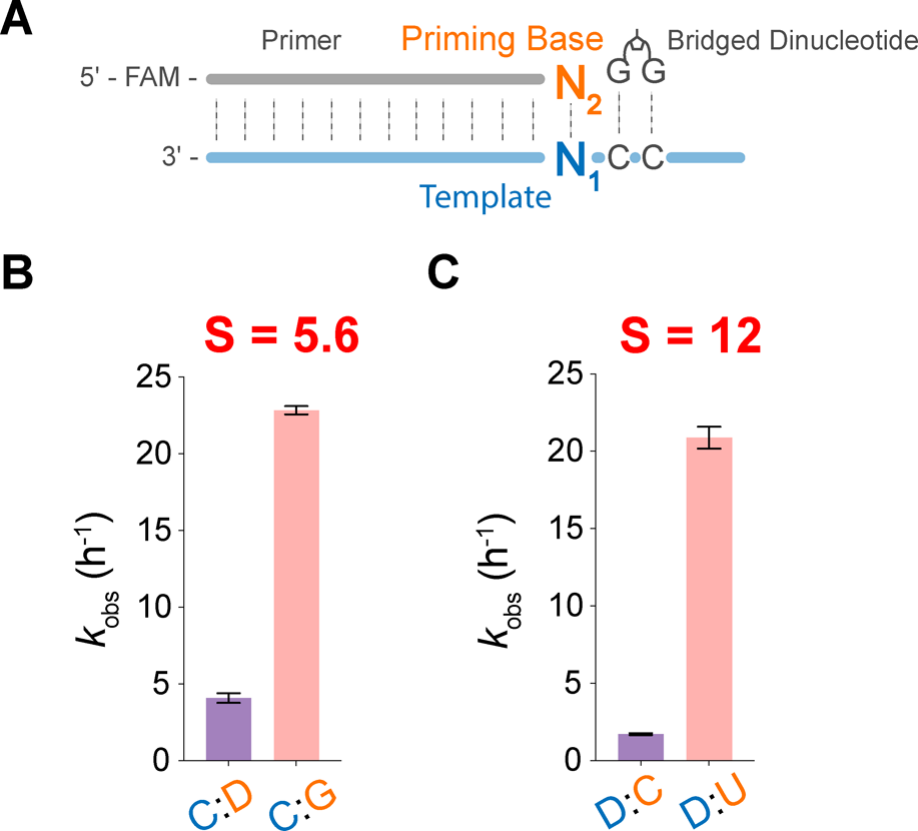
Stalling effects of D:C mismatch. (A) Schematic representation of the primer extension reactions for evaluating the stalling effects of terminal D:C mismatch pairs. (B) Barplot of primer extension reactions (N_1_:N_2_ = C:D and C:G). Stalling factor *S* was calculated from *k*_obs_C:G_/*k*_obs_C:D_. (C) Barplot of primer extension reactions (N_1_:N_2_ = D:C and D:U). Stalling factor *S* was calculated from *k*_obs_D:U_/*k*_obs_D:C_. Primer extension reactions were performed at 10 mM bridged dinucleotide (G*G), 1.5 μM primer, 2.5 μM template, 100 mM MgCl_2_, and 200 mM Tris at pH 8.0. Error bars indicate standard deviations of the mean, *n* = 4 replicates.

To address the concern raised by the elevated formation rates of D:C pairs, we investigated the influence of terminal D:C mismatches on subsequent nonenzymatic template primer extension. We prepared template-primer duplexes with either a D:C, D:U, or C:G base pair at 3′-end (Table S5) and measured their primer extension rates. We observed that the incorporation of nucleotides following a terminal D:C mismatch is significantly slower than following the complementary base pairs (C:G and D:U) (Figure 4). Therefore, D:C mismatches have a strong stalling effect on downstream primer extension. We quantified this impact using the stalling factor S, defined as the ratio of rates associated with terminal complementary and mismatched base pairs, *k*_obs_C:G_/*k*_obs_C:D_ and *k*_obs_D:U_/*k*_obs_D:C_. The stalling factors are 5.6 and 12, respectively (Figure 4B,C). This significant stalling effect hinders primer extension after D:C misincorporations and therefore enhances the overall fidelity of template copying in the noncanonical DUCG system.

#### Crystal Structures of RNA Duplexes Containing D:U and D:C Pairs

To further understand the structure and properties of the D:U base pair and the D:C mismatch, we designed and synthesized self-complementary RNA sequences that form duplexes with two separated or adjacent D:U or D:C pairs (Table S6). The sequence UD-1, 5′-AGA GDA GAU CUU CUC U-3′, can form two separated D:U pairs with the underlined nucleobases, while the sequence CD-1, 5′-AGA GDA GAU CUC CUC U-3′, can form two separated D:C pairs. Similarly, the sequence UD-2, 5′-AGA GAA GDU CUU CUC U-3′, can form two adjacent D:U pairs with the underlined nucleobases, while the sequence CD-2, 5′-AGA GAA GDC CUU CUC U-3′, can form two adjacent D:C pairs. All four oligonucleotides crystallized within 2–3 days at 20 °C under their optimal crystallization conditions (Table S7), and we solved their structures by X-ray diffraction at a resolution higher than 1.65 Å. Data collection and structure refinement statistics are summarized in Tables S8 and S9. We found that all four structures adopt the same space group (*R*32). Each unit cell contains only a single RNA strand so that each duplex features two identical D-containing base pairs.

**Figure 5:**
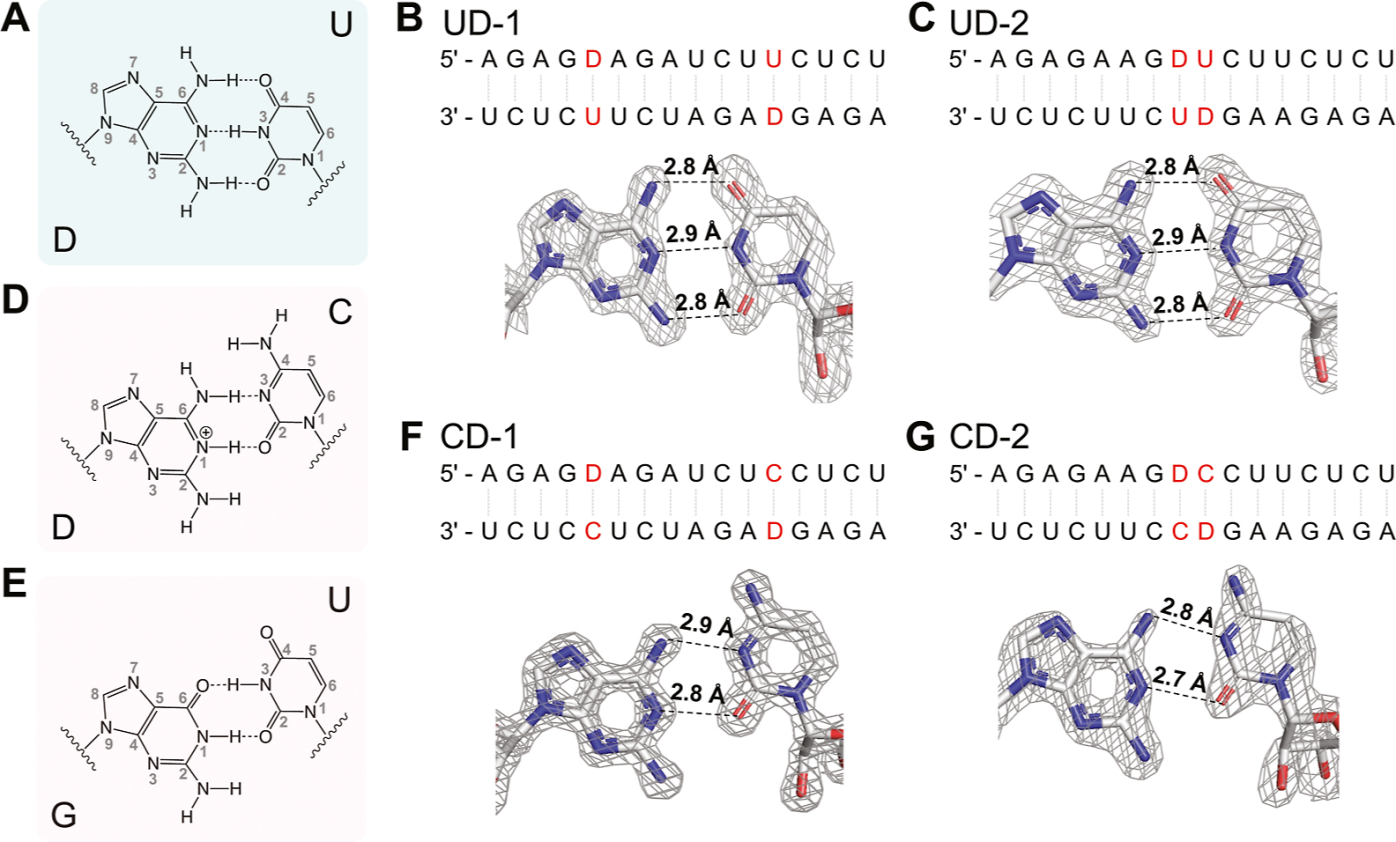
Crystal structures of D:U and D:C pairs. (A) Chemical structure of the complementary D:U pair. (B) Sequences and crystal structures of the UD-1 duplex containing two separated D:U pairs. (C) Sequences and crystal structures of the UD-2 duplex containing two adjacent D:U pairs. (D) Chemical structure of the mismatched D:C pair. (E) Chemical structure of the G:U wobble pair. (F) Sequences and crystal structures of the CD-1 duplex containing two separated D:C pairs. (G) Sequences and crystal structures of the CD-2 duplex containing two adjacent D:C pairs. The gray meshes indicate the corresponding 2F_o_–F_c_ omit maps contoured at 1.5 σ.

Our crystallographic studies show that the D:U base pair has the expected Watson–Crick geometry. Whether separated (UD-1) or adjacent (UD-2) within the RNA duplex, the D:U pairs have identical geometries and exhibit three hydrogen bonds (Figure 5A). Relative to a canonical A:U base pair, the third hydrogen bond is between the exocyclic 2-amino group and O2 of U. The H-bond distances between N_6_–O_4_, N_1_–N_3_, and N_2_–O_2_ in both D:U pairs are identical: 2.8, 2.9, and 2.8 Å, respectively (Figure 5B,C). X-ray crystal structures of DNA duplexes containing D:U pairs reveal their tendency to adopt a Z-form structure.^39,40^

Examination of the crystal structures of the CD-1 and CD-2 duplexes shows that the D:C mismatches all adopt the classical wobble base pair geometry that is isomorphic with a G:U wobble pair (Figure 5D,E). The N_6_–N_3_ and N_1_–O_2_ distances are similar for CD-1 and CD-2 duplexes: 2.9 Å vs 2.8 and 2.8 Å vs 2.7 Å (Figure 5F,G). Based on the observed interatomic distances, the D:C pairs in the CD-1 and CD-2 duplexes appear to have two hydrogen bonds: N_6_–N_3_ and N_1_–O_2_. However, an N_1_–O_2_ hydrogen bond requires N_1_-protonation of D in D:C pairs. Solution-phase NMR studies revealed that the 2-aminopurine (2AP)-cytosine (C) pair exists predominantly as a protonated pair as opposed to an imino tautomer at physiological pH.^41^ Since D has an additional electron-donating amino group, N_1_ in D has a higher p*K*_a_ value and is more likely to be protonated than N_1_ in 2AP.^42^ Therefore, D in the D:C pairs likely exists as a protonated D in the canonical amino tautomeric form (Figure 5D).

### Sequencing Analysis of the AUCG (Canonical) and DUCG (Noncanonical) Systems

**Figure 6:**
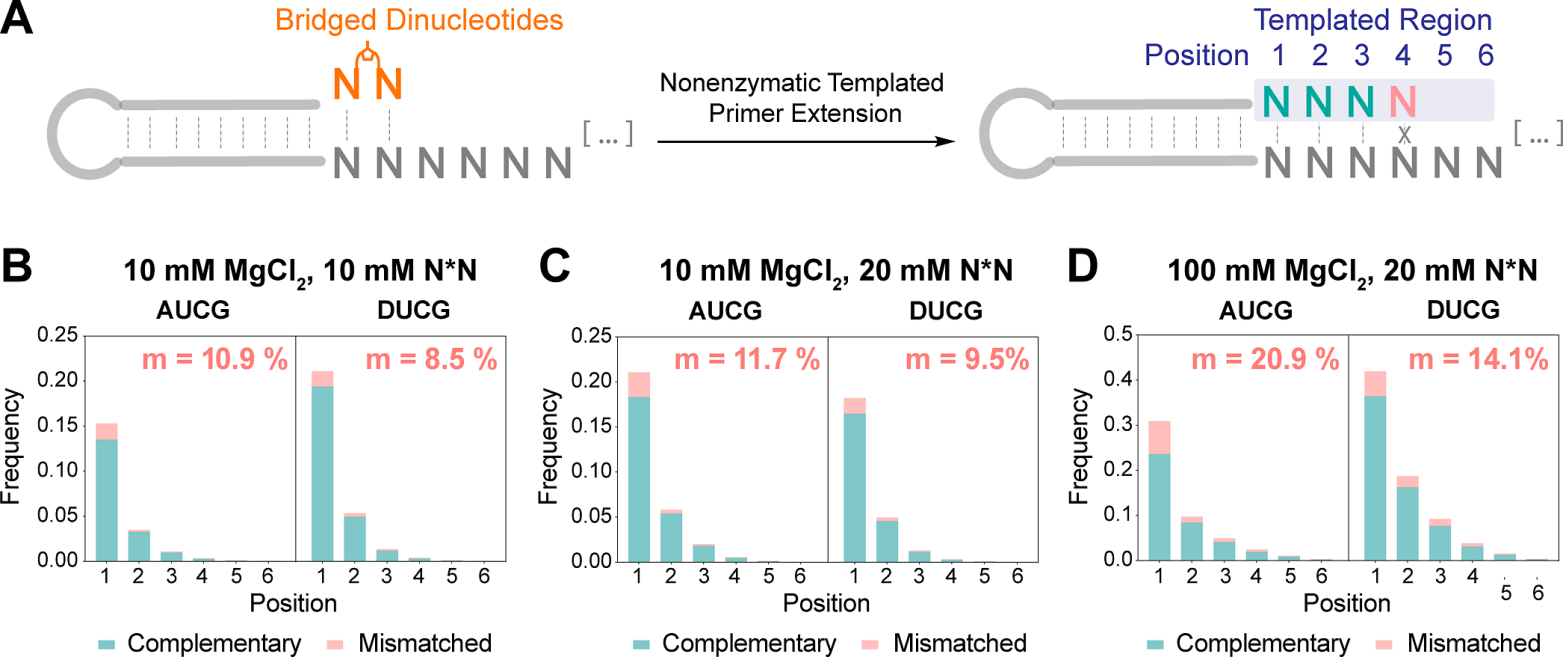
(A) Schematic representation of primer extension on a hairpin primer/template. (B–D) Yield and fidelity stacked barplots show the position-dependent complementary and mismatched incorporation frequency in AUCG and DUCG systems. At each position, the sum of the frequencies of primer extension with a complementary nucleotide, a mismatched nucleotide, and no extension was normalized to 1. Mismatch frequency (*m*) is calculated for the extended sequence at position 1–4. Stacked barplots are generated for the following reaction conditions: (B) 10 mM MgCl_2_ and 10 mM N*N, (C) 10 mM MgCl_2_ and 20 mM N*N, and (D) 100 mM MgCl_2_ and 20 mM N*N. Reactions conditions: 1 μM hairpin, 1.2 μM 5′ handle block, 200 mM Tris at pH 8.0, 100 mM/10 mM MgCl_2_, 20 mM/10 mM N*N, incubated at RT for 24 h.

We next conducted competition experiments in which all four activated monomers were used to copy a random sequence template region. We then used deep RNA sequencing to compare the efficiency and fidelity of the noncanonical DUCG system to the canonical AUCG system in the nonenzymatic primer extension. We adapted the protocol for Nonenzymatic RNA Primer Extension Sequencing (NERPE-Seq):^36^ two sets of mixed bridged dinucleotides (N*N, where N = A, U, C, and G in the AUCG system and N = D, U, C, and G in the DUCG system) were added to the respective self-priming hairpin constructs (Table S10) with a 6-nucleotide long randomized region containing all four bases (6N, where N = A, U, C, and G in the AUCG system and N = D, U, C, and G in the DUCG system). The presence of templating and extended nucleotides within the same read allowed determination of the fidelity of the copied products. The reaction mixtures were incubated for 24 h then quenched and processed for next-generation sequencing (Figure 6A). We used Moloney Murine Leukemia Virus (MMLV) Reverse Transcriptase for cDNA synthesis, which has been reported to incorporate T opposite D with high fidelity.^43^ We then examined the resulting sequences to determine the yield, fidelity, product distribution, and mismatch patterns of the products of nonenzymatic copying.

#### Yield and Fidelity

We determined the extent and fidelity of template-directed primer extension following a single addition of activated substrates to the primer/template hairpin construct. We used three different reaction conditions to investigate the influence of MgCl_2_ and bridged dinucleotide (N*N) concentration: 10 mM MgCl_2_, 10 mM N*N; 10 mM MgCl_2_, 20 mM N*N; and 100 mM MgCl_2_, 20 mM N*N. Each individual product sequence was categorized as complementary, mismatched, or unextended. We then generated stacked barplots with position-dependent frequencies of complementary and mismatched incorporation. Mismatch frequency (*m*) was computed as the fraction of mismatched over total incorporations across all positions.

Our comparative analysis reveals that the DUCG system exhibits a modest improvement over the canonical AUCG system in both yield and fidelity. This improvement is most notable at lower MgCl_2_ and subsaturating substrate concentrations. The DUCG system exhibits a higher frequency of position-dependent incorporation under most of the tested reaction conditions (Figure 6B–D). The system also has a higher frequency of total extended products: a 38% increase in 10 mM MgCl_2_, 10 mM N*N and a 36% increase in 100 mM MgCl_2_, 20 mM N*N (Table S11). Furthermore, an improvement in fidelity is observed across all reaction conditions in the DUCG system, with a notable 20–30% decrease in error rate compared to the AUCG system (Figure 6B–D). The fidelity advantage of the DUCG system is most apparent at high MgCl_2_ concentration, which catalyzes the hydrolysis of bridged dinucleotides, thereby reducing the bridged-to-mononucleotide ratio and consequently decreasing fidelity.^5^ In addition, the mismatch ratio is not affected by the concentration of bridged dinucleotides, as the bridged-to-mononucleotide ratio is independent of the substrate concentration. Overall, the elevated yield and increased fidelity observed in the DUCG system affirm its advantage in nonenzymatic template copying.

#### Distribution of Product Bases

**Figure 7:**
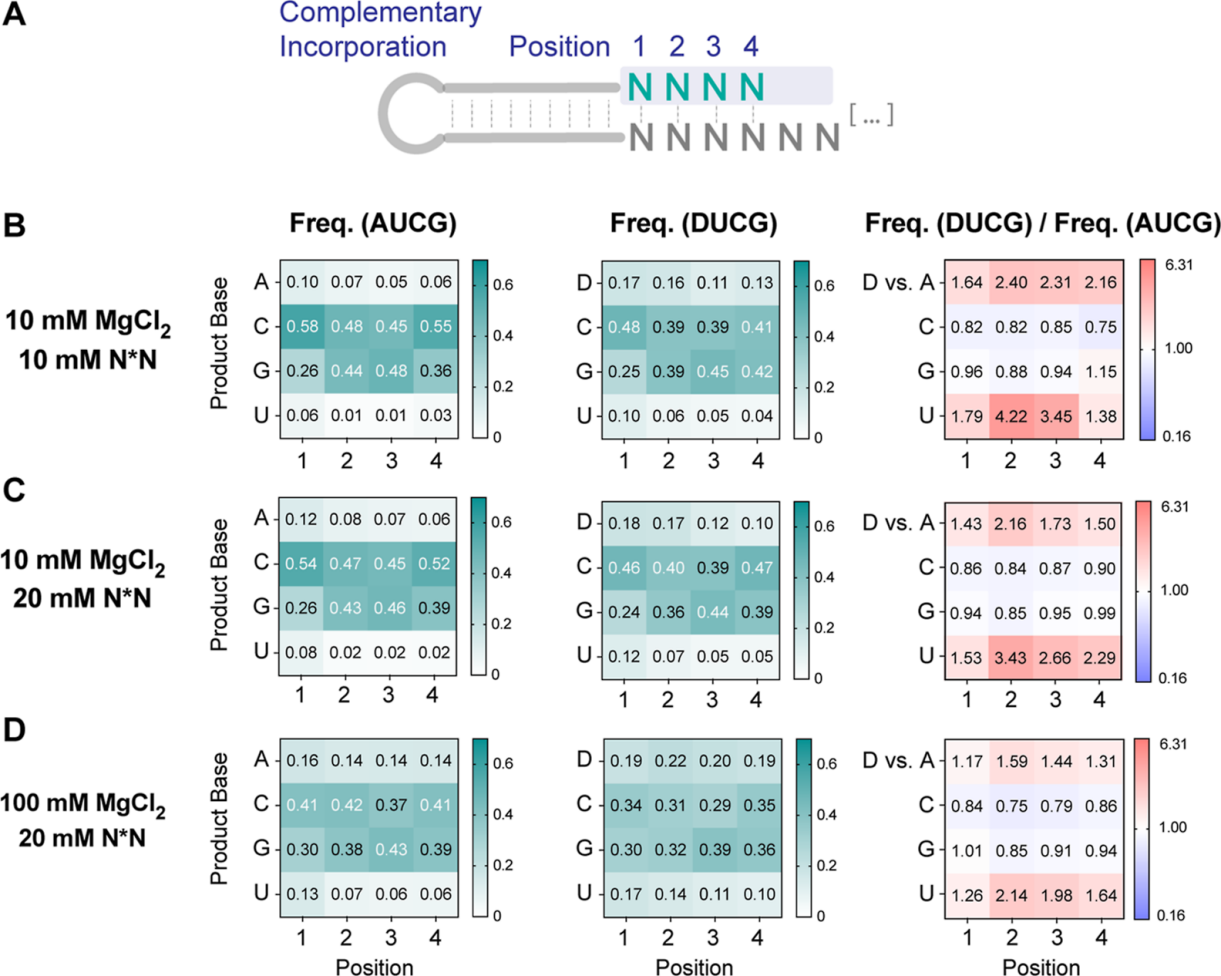
DUCG system decreases biases in complementary product incorporation by enriching D and U in the product base distribution. (A) Schematic representation of the product base distribution. (B–D) Position-dependent product base frequency in the AUCG and DUCG systems and the frequency ratio between AUCG and DUCG. Heatmaps are generated for the following reaction conditions: (B) 10 mM MgCl_2_ and 10 mM N*N, (C) 10 mM MgCl_2_ and 20 mM N*N, and (D) 100 mM MgCl_2_ and 20 mM N*N. For the frequency ratio heatmap, red represents greater frequency in the DUCG system, whereas blue represents greater frequency in the AUCG system.

After probing the yield and fidelity of the AUCG and DUCG systems, we next examined the product composition. Nonenzymatic primer extension products generated by the addition of an equimolar mixture of all four activated canonical nucleotides tend to be rich in G and C, in part because the A:U base pair is weaker than the G:C pair.^5^ We wondered whether the DUCG system could alleviate this biased incorporation. We quantified the product base distribution among fully complementary products for both the AUCG and DUCG systems (Figure 7). To better compare the two systems, we also plotted the ratio of frequencies between the DUCG and the AUCG systems. Numbers above 1 (colored in red) indicate enrichment and those below 1 (colored in blue) indicate diminishment in the DUCG system. The product base distribution of the AUCG system across all reaction conditions is heavily biased toward G and C incorporation, aligning with findings from prior research.^5^ In contrast, the DUCG system has a more even product base distribution with a significant increase in the incorporation of both D and U. For example, under the 10 mM MgCl_2_ and 10 mM N*N condition, product base frequency of D, compared to A, increased from 0.10 to 0.17 and that of U increased from 0.06 to 0.10 in position 1. This phenomenon is especially significant at lower MgCl_2_ concentrations, as the effect of enhanced affinity of the D:U base pair becomes more dominant. Overall, the DUCG system partially mitigates the issue of biased incorporation, although even in the DUCG system, the incorporation of G and C is 2 to 4-fold greater than that of D and U.

Beyond examining the product base distribution, we delved into the distribution of inferred bridged dinucleotides, which serve as the primary substrates for template copying. Leveraging the template composition information made possible by deep sequencing, we deduced the normalized distribution of the 16 possible bridged dinucleotides involved in the nonenzymatic primer extension. In alignment with previous research,^5^ the AUCG system exhibited an increased frequency of G and C in both the first and second positions of the inferred bridged dinucleotides (Figure S1), attributed to their enhanced binding affinity with the template. We were gratified to see that the DUCG system substantially mitigated this bias by enhancing the incorporation of D*N and U*N 2 to 8-fold across all reaction conditions (Figure S1B–D). The increased incorporation frequency observed with D*N and U*N approximately doubles when the second nucleotide is also a D or U (i.e., D*D, D*U, U*D, and U*U). This outcome underscores the impact of the enhanced binding affinity of D on the bridged dinucleotide primer extension pathway (Figure 1C). It serves as an effective mechanism for equalizing the product distribution in nonenzymatic primer extension processes, thereby maximizing the diversity of inherited genetic information.

#### Mismatch Composition and Stalling

We measured the position-dependent frequency of all 12 possible mismatches in the AUCG and DUCG systems. Consistent with previous research,^5^ the A:G and D:G (template: product) pair is the most frequent mismatch at position 1 in both systems across all reaction conditions (Figure S2). Interestingly, the D:C and C:D mismatches are not significantly overrepresented when compared with A:C and C:A mismatches.

**Figure 8:**
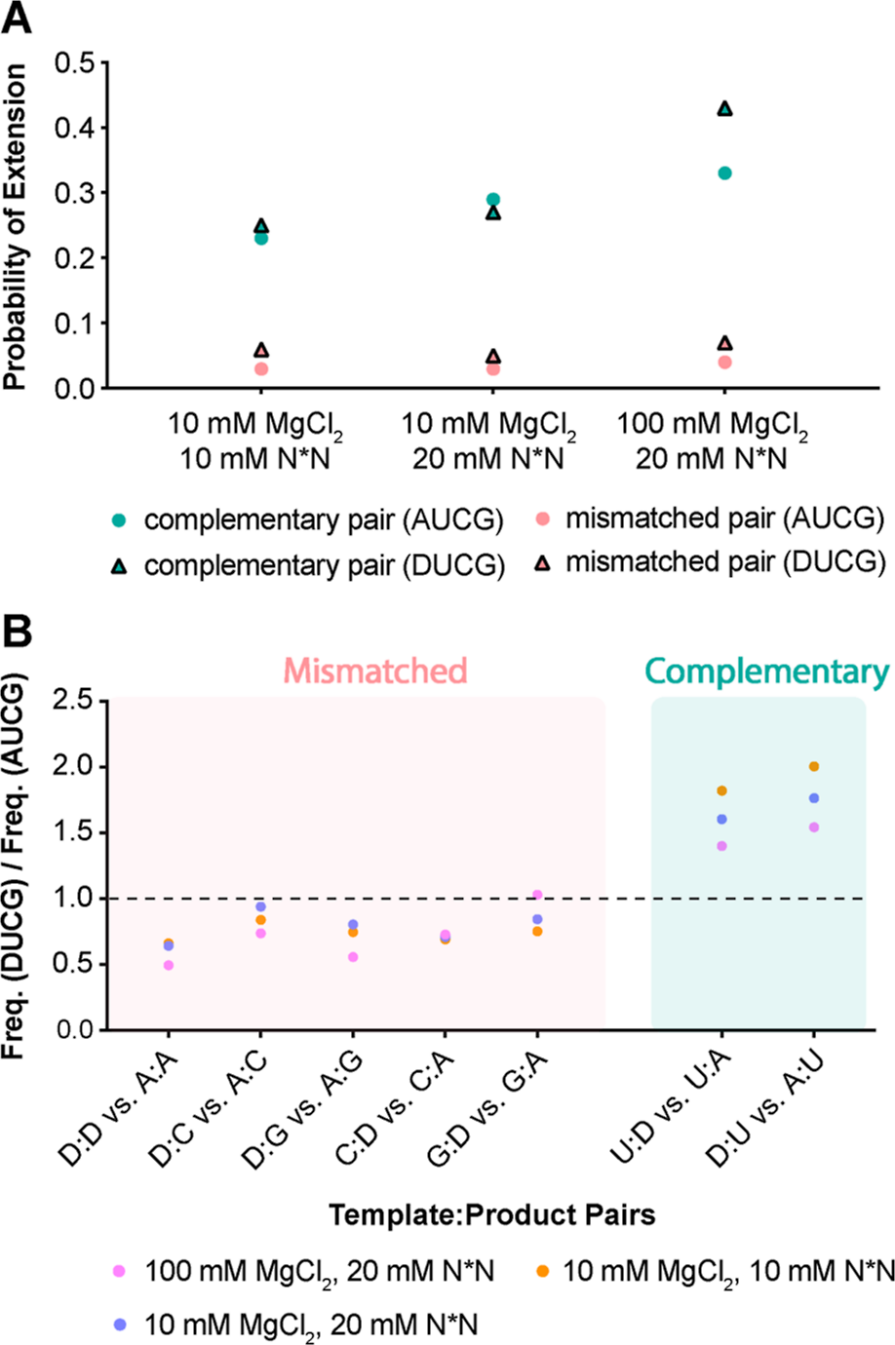
Effect of mismatches on subsequent primer extension and the impact of D substitution. (A) Probability of extension following a complementary pair versus a mismatched pair at position 1 in the AUCG and DUCG systems. (B) Frequency ratio of overall T:P pair distribution among all incorporation (mismatched incorporation + complementary incorporation) between the DUCG and AUCG systems. Frequency ratios of T:P pairs containing D are selected and plotted.

The deep sequencing data also allowed us to look at the effect of mismatches on subsequent primer extension. In both systems, the probability of primer extension past a mismatched pair at position 1 is significantly lower compared with that past a complementary pair (Figure 8A). The complementary pairs exhibit a 4 to 9-fold higher likelihood of extension compared to mismatched pairs under all tested reaction conditions. This indicates that once a mismatch is incorporated, it is less likely to extend further than a matched nucleotide, as expected from the stalling effect we observed previously for D:C mismatch pairs. Furthermore, we quantified the extension probability over each type of mismatch at the +1 position (Figure S3A) along with the corresponding stalling factor (Figure S3B). The stalling factors range from roughly 2 to 10 depending on the mismatch.

The error frequency for primer extension after a mismatch is also significantly higher than the overall mismatch frequency in both systems and across all reaction conditions (Figure S4). The mismatched priming base pair seems to lack the proper conformation required to enforce correct base pairing on the subsequent nucleotide incorporation. Overall, all template:product (T:P) pairs containing D display a decrease in subsequent mismatched pair frequency and a corresponding increase in complementary frequency (Figure 8B), underscoring the advantage of the DUCG system in the fidelity of nonenzymatic template copying.

## Discussion

To ensure the efficient transmission of genetic information before the advent of ribozymes, we examined modifications of the A:U base pair that would address the issue of incorporation biases in nonenzymatic RNA template copying. Our previous study demonstrated that the A:s^2^U (2-thio-U) pair stabilizes RNA duplexes by reducing the desolvation penalty and preorganizing single-stranded RNA during hybridization.^38^ In this study, we evaluated the alternative D:U base pair, which enhances binding through an additional hydrogen bond and stronger stacking interactions. As a result, we observed a 20-fold increase in the binding of the substrate (D*D vs A*A) to a −UU– template (Figure 2). Accordingly, the advantage of D is less pronounced at higher substrate concentrations (Figures 2 and 3) and in the presence of a downstream activated trimer helper (Figure 3). The latter reacts with an activated mononucleotide to form a highly reactive monomer-bridged-trimer intermediate,^34^ masking the advantage of D’s increased binding strength. These results collectively suggest that the distinctive benefit of D is most notable at low and subsaturating substrate concentrations.

We examined the D system in both a defined construct and in competition experiments where all four nucleotides vied for incorporation on a mixed-sequence template. The most notable difference is the drastic rate increase for C:D (12-fold) and D:C (7.7-fold) mismatches in the defined construct (Figure 3B), in contrast to their marginal (Figure S2) or reduced (Figure 8B) presence in the competition experiments. Crystallographic studies reveal that the protonated amino form of D can form a base pair with C that is isostructural to a G:U wobble pair (Figure 5), which may account for the increased rate of D:C mismatches in the defined construct (Figure 3B). In competition experiments, D:C mismatches are likely to be outcompeted by the correct C:G and D:U pairs during incorporation, accounting for their infrequent occurrence (Figure S2). Additionally, the strong stalling effects of the D:C mismatches (Figures 4 and S3B) further reduce concerns regarding the fidelity of the DUCG system.

In addition to the inhibitory effect of the D:C mismatches on subsequent primer extension, our competition experiments showed a pronounced stalling effect across all other mismatched pairs, effectively hindering downstream primer extension and therefore improving the system’s overall fidelity.^44^ The A:G and D:G (template: product) mismatches emerged as the most significant among all 12 mismatches studied (Figure S2). The higher prevalence of A:G and D:G mismatches as compared to G:A and G:D mismatches most likely stems from the effective G template depletion by the correct C incorporation.^5^ We were pleased to find that the A:G and D:G mismatches have the lowest probability of extension (Figure S3A) and the highest stalling factor (Figure S3B). Furthermore, mismatches that extend are more susceptible to further misincorporations (Figure S4), likely due to the lack of conformational rigidity in the priming base pair. Overall, the probability of extension over a mismatched pair is much lower than that of a complementary pair in both systems (Figure 8A), effectively reducing downstream error propagation and enhancing the overall fidelity.

Through deep sequencing, we observed that the DUCG system outperforms the AUCG system in both yield and fidelity (Figure 6). This effect is most notable at low MgCl_2_ and substrate concentrations, which are more compatible with the fatty acid-based membranes^45^ and which may be more prebiotically plausible.^46,47^ Moreover, the copying of template nucleotides is more uniform in the DUCG system under all tested reaction conditions (Figures 7 and S1). Despite improvements, the G and C-bias issue still persists (Figure 7), since the D:U base pair is not as strong as the G:C base pair. This is likely attributable to the repulsive electrostatic cross-interactions between the functional groups in the D:U pairs,^31^ as supported by thermodynamic^12,48^ and computational studies.^11^

Lastly, although our work is part of a larger effort to examine the origins of the RNA World, the implications of our research extend beyond the origins of life field. By experimentally characterizing the enhanced binding affinity of the D:U pair, we highlighted a chemical means of strengthening RNA–RNA interactions. In addition, we showed that the D:C mismatches adopt the classical wobble base pair geometry. Furthermore, we established an RNA sequencing and bioinformatics analysis pipeline to probe the effect of single or multiple nucleotide changes to the canonical 4-letter AUCG genetic system.

## Conclusions

In summary, diaminopurine (D) has emerged as a promising primordial nucleobase candidate, showing advantageous attributes in the nonenzymatic primer extension within the DUCG system. With an extra hydrogen bond and increased stacking interactions, D:U pairs exhibit enhanced copying at low substrate concentrations, as reflected by a significant reduction in *K*_m_. Furthermore, while D:C mismatches are significant in a defined construct, they do not compromise the system’s fidelity in a competition setting. Moreover, they, along with other mismatches, demonstrate a strong stalling effect on primer extension. Thus, the DUCG system outperforms the canonical AUCG system in both yield and fidelity, especially at lower MgCl_2_ and substrate concentrations. We may therefore ask whether the DUCG system is a plausible primordial genetic alphabet. The answer to this question depends primarily on whether a high yielding and potentially prebiotic synthesis of D nucleotides can be discovered. A second key question is whether the greater RNA duplex stability that results from replacing A with D is an advantage or a disadvantage in terms of nonenzymatic RNA replication. Further research into DUCG and other potential primordial genetic alphabets will be required to address these questions.

## Data Availability

Raw sequencing files, processing worksheets, and code can be found in the OSF.io repository: https://osf.io/zscy8/.

## Supporting Information Available

Materials and methods; RNA nearest neighbor energy prediction; bridged dinucleotides distribution heatmaps; mismatch heatmaps; stalling effect of mismatches; extending mismatch stacked barplots; sequences of the primer, template, blocker, and complementary oligonucleotide used in the Michaelis-Menten reactions, primer extension reactions, and stalling effect experiments; RECCES-Rosetta predictions, experimentally reported NN parameters, and MD/QM predictions; stacking interaction energy differences predicted by the MD/QM calculations; sequences of the self-complementary RNA duplexes used in crystallographic studies; optimized conditions for crystallization; data collection statistics; structure refinement statistics; and sequences of the template and complementary oligonucleotides used in the deep sequencing experiments and overall frequencies of total extended products in AUCG and DUCG systems and their ratios (PDF)

## Abbreviations

D: diaminopurine
NN: nearest-neighbor
MD/QM: molecular dynamics/quantum mechanics
